# Comprehensive analysis of the laccase gene family in tea plant highlights its roles in development and stress responses

**DOI:** 10.1186/s12870-023-04134-w

**Published:** 2023-03-07

**Authors:** Jiaxin Zhu, Hongxiu Zhang, Kelin Huang, Rui Guo, Jingjuan Zhao, Hui Xie, Junyan Zhu, Honglian Gu, Hongrong Chen, Guoqiang Li, Chaoling Wei, Shengrui Liu

**Affiliations:** 1 State Key Laboratory of Tea Plant Biology and Utilization, Key Laboratory of Tea Biology and Tea Processing of Ministry of Agriculture and Rural Affairs, Anhui Agricultural University, 130 Changjiang West Road, Hefei, 230036 China; 2 Lu’an Institute of Product Quality Supervision and Inspection, Lu’an City, China

**Keywords:** Laccase, Tea plant, Development, (A)biotic stress, Expression patterns, SSR markers

## Abstract

**Background:**

Laccase (LAC) is the pivotal enzyme responsible for the polymerization of monolignols and stress responses in plants. However, the roles of LAC genes in plant development and tolerance to diverse stresses are still largely unknown, especially in tea plant (*Camellia sinensis*), one of the most economically important crops worldwide.

**Results:**

In total, 51 *CsLAC* genes were identified, they were unevenly distributed on different chromosomes and classified into six groups based on phylogenetic analysis. The *CsLAC* gene family had diverse intron–exon patterns and a highly conserved motif distribution. *Cis*-acting elements in the promoter demonstrated that promoter regions of *CsLACs* encode various elements associated with light, phytohormones, development and stresses. Collinearity analysis identified some orthologous gene pairs in *C. sinensis* and many paralogous gene pairs among *C. sinensis*, *Arabidopsis* and *Populus*. Tissue-specific expression profiles revealed that the majority of *CsLACs* had high expression in roots and stems and some members had specific expression patterns in other tissues, and the expression patterns of six genes by qRT‒PCR were highly consistent with the transcriptome data. Most *CsLACs* showed significant variation in their expression level under abiotic (cold and drought) and biotic (insect and fungus) stresses via transcriptome data. Among them, *CsLAC3* was localized in the plasma membrane and its expression level increased significantly at 13 d under gray blight treatment. We found that 12 *CsLACs* were predicted to be targets of cs-miR397a, and most *CsLACs* showed opposite expression patterns compared to cs-miR397a under gray blight infection. Additionally, 18 highly polymorphic SSR markers were developed, these markers can be widely used for diverse genetic studies of tea plants.

**Conclusions:**

This study provides a comprehensive understanding of the classification, evolution, structure, tissue-specific profiles, and (a)biotic stress responses of *CsLAC* genes. It also provides valuable genetic resources for functional characterization towards enhancing tea plant tolerance to multiple (a)biotic stresses.

**Supplementary Information:**

The online version contains supplementary material available at 10.1186/s12870-023-04134-w.

## Introduction

The tea plant (*Camellia sinensis* (L.) O. Kuntze) is one of the most important woody cash crops, which tender buds and leaves are the raw material for the most widely consumed non-alcoholic beverages worldwide [1, 2]. The stems and leaves of tea plants have excellent physical and mechanical properties. The structure of the cell wall, which consists of cellulose, hemicellulose, pectin, protein and lignin, is one of the most pivotal contributing factors to these properties [3]. With the published genome of the tea plant [4, 5], genome-wide analysis of genes encoding enzymes participate in the lignin biosynthesis can be implemented. Studies have revealed that PAL, C4H, C3H, 4CL, HCT, CCR, CCoAOMT, CAD, F5H and COMT participate in lignin biosynthesis in plants [6–9]. However, how laccase is involved in lignin biosynthesis in tea plants remains unclear.

Lignin is the second most abundant biopolymer, and primarily consists of three canonical monomers, namely coniferyl (G), sinapyl (S) and p-coumaryl (H) alcohols [10]. Lignin monomers are synthesized in the cytosol and then exported to the apoplastic region, followed by oxidation and polymerization into lignin through a random coupling process [11]. During the polymerization process, laccase (p-diphenol:dioxygen oxidoreductase, EC 1.10.3.2) is the critical enzyme implementing single electron oxidation of phenolic compounds generating resonance structures [12]. Laccase is the largest subfamily of multicopper oxidases (MCOs), which have three conserved catalytic sites (Cu-oxidase, Cu-oxidase_2, and Cu-oxidase_3) that combine with four copper (Cu) ions and have a wide range of substrates. Laccases are widely present in bacteria, fungi, insects and plants, and many studies have shown the role of laccases in lignin biosynthesis and stress responses in plants.

Earlier studies have verified that many laccases can catalyze the oxidative polymerization of lignin precursors [13, 14]. Subsequent studies have further confirmed that laccase genes play crucial roles in the biosynthesis of lignin in some model and economically important plants, such as *Arabidopsis thaliana* [15], *Brachypodium distachyon* [16], *Oryza sativa* [17], *Cleome hassleriana* [18], *Pyrus bretschneideri* [19], and *Phyllostachys edulis* [11]. Among the 17 *AtLACs* in *Arabidopsis thaliana*, both *AtLAC4* and *AtLAC17* contribute to the constitutive lignification of stems, and lignin contents were slightly decreased in the double mutants *Atlac4 lac11* and *Atlac4 lac17*; *AtLAC11* was also found to participate in lignin polymerization, and the lignin content was tremendously decreased in the triple mutant *Atlac4 lac11 lac17* [20]. Liu et al. (2017a) found that *OsLAC10* was not only involved in lignin biosynthesis but also participated in the copper stress response in *Oryza sativa*. In the seed coats of *Cleome hassleriana,* ChLAC8 was essential for catechyl lignin polymerization and determined the lignin composition when caffeyl alcohol was available. In *Gossypium hirsutum*, overexpression of *GhLac1* enhanced broad-spectrum biotic defense responses to both pathogens and pests by increasing lignin deposition [21]. The identified *PeLAC10* in *Phyllostachys edulis* was overexpressed in *Arabidopsis*, demonstrating that the lignin content was increased and the adaptability to phenolic acid and drought stresses were improved in transgenic *Arabidopsis* [11]. Overall, these studies have shown that laccases play a pivotal role in plant development and responses to stresses by mediating lignin biosynthesis.

To clarify the role of the laccase genes in lignin biosynthesis in tea plant, we comprehensively analyzed the *CsLAC* gene family. Our analyses included determining chromosomal locations, evolutionary relationships, collinearity, gene structures and conserved motifs, *cis*-acting elements, protein interaction networks, target gene prediction of miR397, tissue-specific expression patterns, and expression profiles in response to biotic and abiotic stresses, as well as the development of polymorphic SSR markers. This study provides an important basis for further investigation of the role of *CsLACs* in the regulation of lignin biosynthesis and stress tolerance in tea plants.

## Materials and methods

### Plant material

Eight different tissues, including the apical bud, first leaf, second leaf, third leaf, young stem, young root, budding flower in autumn, and young fruit in summer, were collected from the 6-year-old cloned tea cultivar ‘Shuchazao’, which was planted in the Tea Plant Cultivar and Germplasm Resource Nursery (Hefei, Anhui, China) with good field management [2].

Two-year-old cloned tea plants (*Camellia sinensis* cv. ‘Shuchazao’) were cultured in plastic pots (30 cm diameter and 35 cm height) and grown under controlled conditions (23 ± 3 °C with 65 ± 5% humidity and a 16/8 h day/light photoperiod) at Anhui Agricultural University (Hefei, China). Plants with uniform growth (25–30 cm height) and without signs of disease and insects were used for our experiments. For the insect feeding treatment, tea geometrids (*Ectropis obliqua*) at the 3^rd^ larval stage were starved for 8 h and distributed evenly on the tea plant leaves (leaves were at the same position on each plant, with 20 insects per plant), and then insects were removed after one-third of the leaves were consumed [22]. Leaves from the nontreated tea plants were used as controls. All treated and control leaves were collected at 3, 6, 12, and 24 h. Three biological replicates were harvested for each group of samples. All collected samples were immediately frozen in liquid nitrogen and subsequently stored at − 80 °C for further use.

### Identification of the CsLAC gene family

A total of 17 *Arabidopsis* laccase members containing Cu-oxidase (PF00394), Cu-oxidase_2 (PF07731), and Cu-oxidase_3 (PF07732) domains were obtained [23]. To identify the *CsLAC* gene family in the *Camellia sinensis* ‘Shuchazao’ genome [5], BLASTp was performed using AtLAC protein sequences as queries, and sequences with an *E*-value < 10^–10^ were retained. The obtained candidate sequences with no conserved laccase domain were deleted and gene family identification was performed using the SMART (http://smart.embl-heidelberg.de/) and Pfam (http://pfam.xfam.org/) databases. A total of 51 unique *CsLAC* genes were identified, which were named from *CsLAC1* to *CsLAC51* based on their chromosomal location. The CDs and protein sequences of the 51 *CsLAC* genes are listed in Additional file 1: Table S1. To further explore the characteristics of their domain-containing proteins, the ExPasy program (http://web.expasy.org/protparam/) was used to calculate the molecular weight (MW) and isoelectric point (pI), and the online software Cell-PLoc 2.0 (http://www.csbio.sjtu.edu.cn/bioinf/Cell-PLoc-2/) was used to predict their subcellular localization.

### Chromosomal location, phylogenetic analysis and collinearity analysis

To identify their physical locations, the starting position of each *CsLAC* gene on each chromosome was determined by BlastN searches against the database of the complete tea plant genome [5]. The chromosomal locations of all *CsLAC* genes were confirmed by TBtools software (http://www.tbtools.com/). The amino acid sequences were used to construct phylogenetic relationships with MEGA6.0 using the neighbour-joining method (1000 bootstrap replications) [24]. The collinearity analysis of *LAC* genes within tea plants and among different plant species (*Arabidopsis thaliana*, *Camellia sinensis* and *Populus trichocarpa*) was performed by TBtools using MCScanX software.

### Gene structures, conserved motifs and cis-elements

The exon‒intron structures were determined using the Gene Structure Display Server (http://gsds.gao-lab.org/). The conserved motifs of CsLAC protein sequences were analyzed by the MEME (http://meme-suite.org/tools/meme) program with previously described parameter settings and finally viewed by TBtools [25]. To determine the *cis*-elements, we obtained the 2000-bp sequence upstream from each *CsLAC* initiation codon and predicted their *cis*-elements using the online tool PlantCARE (http://bioinformatics.psb.ugent.be/webtools/plantcare/html/) as described previously [26, 27].

### Protein interaction network analysis

STRING (https://string-db.org/cgi/input.pl?sessionId) [28] was used for protein interaction network analysis and Cytoscape version 3.4.0 was used for construction of corresponding protein‒protein interaction networks.

### Expression patterns of CsLAC genes

The transcriptome data generated from eight tissues, including the bud, the first leaf, the second leaf, the third leaf, stem, root, flower and fruit, were obtained from our previously published RNA-seq data [29]. The transcriptome data of *CsLACs* in response to drought and cold stresses were obtained from previous studies [30, 31]. The RNA-seq data of *CsLACs* in response to fungus and insect stresses were obtained from our previously published data [32] and unpublished data (NCBI SRA: PRJNA901518), respectively.

After obtaining the raw transcriptome data from our previous studies or the SRA database from NCBI, we converted the sra files to fastq format by the SRA Toolkit with fastq-dump and –split-3 parameters. Trimmomatic software was used to filter all raw reads based on standard criteria. The details are as follows: remove technical sequences, set a 5 bp sliding window from the 5’ end of the read and then remove the windows with an average quality below 20, cut off bases with a threshold lower than 3 at the beginning and end of reads, and retain the filtered reads with lengths greater than 40 bp. The obtained reads were used for comparison and assembly by Hisat2 and StringTie. The TPM (transcripts per million reads) values of *CsLACs* were calculated by StringTie and collated as a reference to evaluate the transcript abundance [33]. Heatmaps were drawn by TBtools software to show the different expression profiles.

### Cs-miR397a targeted gene prediction and their expression profiles

miRNA-targeted gene prediction was performed by the online toolbox psRobot (http://omicslab.genetics.ac.cn/psRobot/). Based on small RNAome and transcriptome data, we analysed the expression patterns of miR397/*CsLACs* in response to gray blight treatment [32]. The expression abundance of miR397a was normalized to one million against the total clean reads in each library with the following formula: TPM = actual count of miRNA/total count of clean reads × 1,000,000 [34]. The differential expression of miR397a was analyzed using Student’s *t*-test, and the threshold for individual time points was set as *P* ≤ 0.05 and log_2_ (fold change) > 1 [32].

### RNA extraction and qRT‒PCR analysis

Total RNA was isolated by the RNAprep Pure Plant Kit (Tiangen, Beijing, China) according to the manufacturer’s instructions. The concentration and integrity of the total RNA were examined using an Agilent 2100 Bioanalyzer. The specific primers were designed by Primer 5.0, and *GAPDH* was used as an internal reference gene based on our previous studies [2, 35]. The relative expression levels of *CsLACs* were determined by qRT‒PCR using SYBR Green Mix (Takara, Dalian, China) on a CFX96 real time detection system (Bio-Rad, USA). The detailed reaction system and procedures were performed according to our previous studies [22, 35]. The fluorescence was detected during the extension step and the specificity of the amplicon for each primer pair was confirmed by melting curve analysis.

All reactions were implemented in three biological replicates, and each replicate was measured in triplicate. The relative gene expression levels were calculated by the 2^−ΔΔCt^ method [36]. The primer pairs of the six *CsLACs* used for qRT‒PCR analysis are listed in Additional file 2: Table S2.

### Subcellular localization of the CsLAC3 protein

The open reading frame (ORF) of *CsLAC3* was amplified by RT‒PCR and cloned into the pCAMBIA1305 vector to construct the fusion protein. The empty vector and constructed plasmids were introduced into EHA105 competent cells for transient expression in *Nicotiana benthamiana* leaves. The tobacco leaves were held at 25 °C in the dark and collected for fluorescence examination at 48 h after infection [35]. GFP signals in the transiently infected leaves were observed using an Olympus FV1000 confocal microscope (Olympus, Tokyo, Japan). The relevant primers are listed in Additional file 2: Table S2.

### SSR identification and primer design

Simple sequence repeats (SSRs) are generally defined as repeats consisting of 2–6 bp motifs. Thus, SSRs with these basic motifs were identified from the *CsLAC* gene family. The minimum repeat unit was defined as 6, 5, 4, 4, and 4 for dinucleotides, trinucleotides, tetranucleotides, pentanucleotides, and hexanucleotides, respectively [37]. Subsequently, oligonucleotide primers were designed for the sequences flanking the SSRs by Primer 5.0 software. Amplicons needed to be 100–400 bp in length, and primers were designed with the following parameters: primer length 20–22 bp, with 20 bp as the optimum; GC content 40–60%, with the optimum value of 50%; and Tm 50–60 °C, with 56 °C as the optimum value.

### SSR genotyping and data analysis

A total of 36 SSR loci from 30 *CsLAC* genes were selected for designing primers. To validate the primers, 45 tea cultivars or varieties were used for PCR amplification and subsequent resolution by electrophoresis. The reaction mixtures, thermocycling conditions and protocols for PCR product separation were performed based on a previous study [38]. The amplified fragments were separated on a 96-capillary automated DNA fragment analyzer (Fragment Analyzer™ 96, Advanced Analytical Technologies, Inc., Ames, IA). The separated DNA bands were visually scored using PROSize™ 2.0 software, which was included in the advanced Fragment Analyzer™ 96 system. Only one or two fragments were collected for each individual [37].

The number of alleles (*Na*), Shannon’s information index (*I*), observed heterozygosity (*Ho*), expected heterozygosity (*He*), genetic diversity (GD) and polymorphism information content (PIC) values were calculated with PowerMarker version 3.25 (http://statgen.ncsu.edu/powermarker/downloads.htm) (Liu and Muse 2005).

## Results

### Identification of the CsLAC gene family in tea plant

To identify the *CsLAC* genes, we used the tea plant reference genome [5] and searched the genome with HAMMER 3.0 software for three conserved cupredoxin domains (Cu-oxidase, Cu-oxidase_2, and Cu-oxidase_3). A total of 51 *CsLAC* genes were identified after elimination of redundant genes with only one or two cupredoxin domains or without integral ORFs. The identified *CsLAC* genes were named *CsLAC1* to *CsLAC51* and were analyzed for their basic characteristics, including the amino acid (aa) length, protein molecular weight (MW), isoelectric point (pI), and subcellular localizations (Table 1). The amino acid length of the 51 CsLAC proteins ranged from 454 (CsLAC4) to 608 aa (CsLAC19), while the MW ranged from 50.18 (CsLAC4) to 67.87 kDa (CsLAC19), and the pI ranged from 5.15 (CsLAC35) to 9.71 (CsLAC40). The prediction of subcellular localization showed that all *CsLAC* genes were located in the cell membrane.

**Table 1:** Characteristics of the 51 identified *CsLAC* genes from tea plant genome

Gene ID	Gene name	CDs (bp)	Protein			Subcellular location
			**Length (aa)**	**MW (kDa)**	**pI**	
CsLAC1	CSS0015036	1668	555	61.11	8.34	CM
CsLAC2	CSS0006495	1722	573	64.69	8.00	CM
CsLAC3	CSS0013963	1617	538	60.32	8.84	CM
CsLAC4	CSS0007266	1365	454	50.18	7.18	CM
CsLAC5	CSS0048739	1617	538	60.50	8.48	CM
CsLAC6	CSS0049574	1623	540	60.62	6.85	CM
CsLAC7	CSS0047304	1716	571	63.46	8.92	CM
CsLAC8	CSS0004662	1716	571	63.46	7.22	CM
CsLAC9	CSS0043918	1707	568	63.74	8.98	CM
CsLAC10	CSS0043663	1776	591	66.75	5.17	CM
CsLAC11	CSS0041657	1725	574	63.76	6.30	CM
CsLAC12	CSS0014129	1590	529	58.15	8.07	CM
CsLAC13	CSS0005481	1674	557	62.16	9.12	CM
CsLAC14	CSS0050170	1701	566	63.08	9.58	CM
CsLAC15	CSS0032027	1740	579	64.13	8.90	CM
CsLAC16	CSS0044116	1698	565	62.36	8.31	CM
CsLAC17	CSS0010479	1698	565	62.15	6.18	CM
CsLAC18	CSS0030703	1698	565	62.41	6.88	CM
CsLAC19	CSS0013370	1827	608	67.87	6.53	CM
CsLAC20	CSS0010391	1701	566	62.78	6.59	CM
CsLAC21	CSS0020412	1710	569	62.32	7.34	CM
CsLAC22	CSS0023848	1710	569	62.25	6.71	CM
CsLAC23	CSS0030904	1647	548	59.86	9.28	CM
CsLAC24	CSS0013475	1719	572	63.50	7.07	CM
CsLAC25	CSS0011889	1641	546	60.87	6.79	CM
CsLAC26	CSS0024850	1641	546	60.79	7.14	CM
CsLAC27	CSS0048339	1599	532	59.38	9.07	CM
CsLAC28	CSS0048878	1602	533	58.49	6.14	CM
CsLAC29	CSS0045107	1698	565	62.46	6.33	CM
CsLAC30	CSS0010920	1686	561	61.99	6.25	CM
CsLAC31	CSS0008882	1695	564	62.71	6.08	CM
CsLAC32	CSS0032874	1701	566	63.19	5.67	CM
CsLAC33	CSS0050404	1641	546	61.75	9.50	CM
CsLAC34	CSS0036236	1638	545	61.15	6.09	CM
CsLAC35	CSS0007135	1665	554	62.20	5.15	CM
CsLAC36	CSS0039645	1701	566	63.18	8.30	CM
CsLAC37	CSS0047533	1704	567	63.08	8.52	CM
CsLAC38	CSS0037353	1500	499	55.12	8.61	CM
CsLAC39	CSS0001101	1695	564	63.11	7.64	CM
CsLAC40	CSS0030617	1728	575	63.89	9.71	CM
CsLAC41	CSS0045289	1653	550	60.65	9.04	CM
CsLAC42	CSS0029337	1728	575	63.65	9.18	CM
CsLAC43	CSS0040822	1743	580	63.83	9.30	CM
CsLAC44	CSS0006379	1743	580	64.23	5.44	CM
CsLAC45	CSS0030509	1692	563	63.15	8.62	CM
CsLAC46	CSS0026359	1776	591	66.19	8.13	CM
CsLAC47	CSS0002431	1761	586	65.76	8.53	CM
CsLAC48	CSS0036218	1764	587	65.69	8.39	CM
CsLAC49	CSS0019151	1725	574	63.73	8.36	CM
CsLAC50	CSS0022921	1770	589	66.30	7.29	CM
CsLAC51	CSS0028888	1701	566	63.48	8.62	CM

Note: *bp* base pair, *aa* amino acid, *MW* molecular weight, *pI* isoelectric point, *CM* cell membrane

### Chromosomal distribution and phylogenetic analysis

**Fig. 1:**
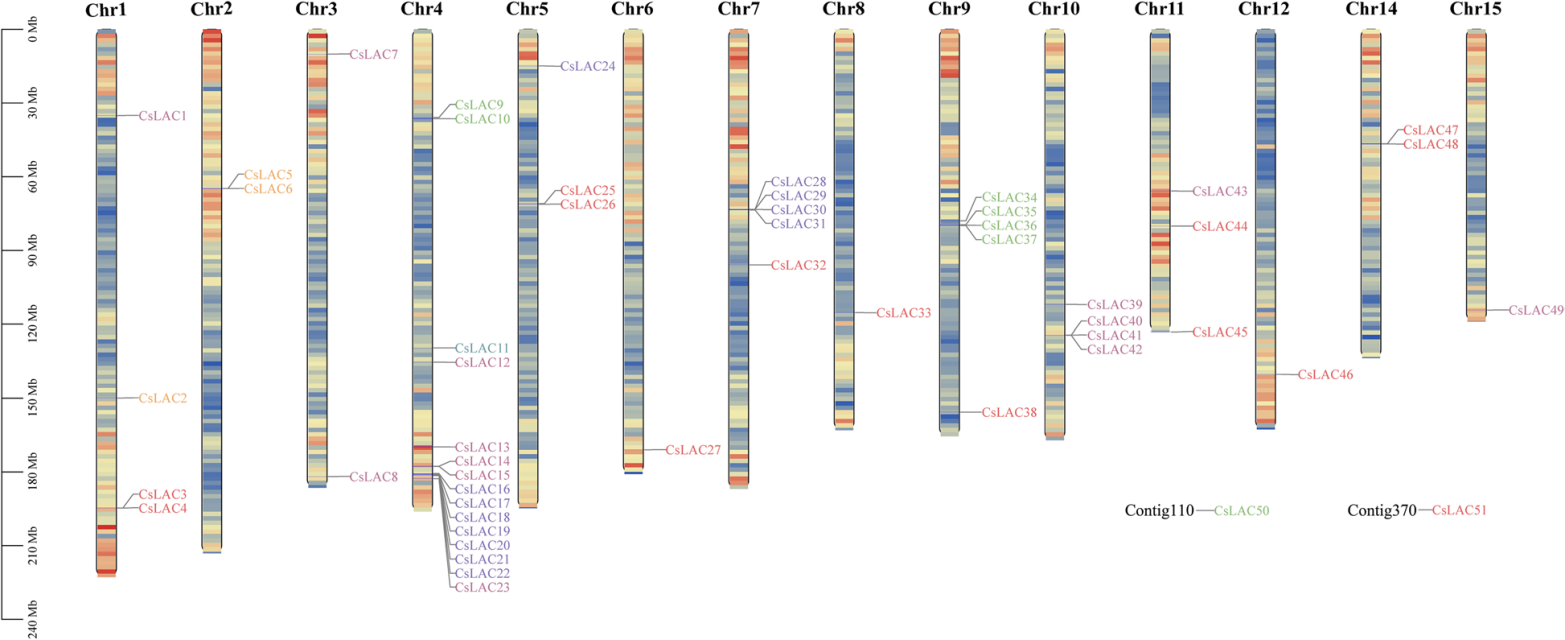
Chromosomal distribution of *CsLAC* family genes in the tea plant genome. The chromosomal position of each *CsLAC* gene was mapped based on the tea plant genome. The ruler on the left represents the physical map distance (Mb). Chromosome 1–15 are arranged from left to right, and two contigs are located on the bottom right corner

The 49 identified *CsLAC* genes were unequally mapped onto 14 out of 15 chromosomes, while the chromosomal locations of the remaining 2 *CsLAC* genes were on unassigned contigs (Fig. 1). Among these chromosomes, Chr4 had the highest number of *CsLAC* genes, with a total of 15 members (*CsLAC9* to *CsLAC23*). However, Chr6, Chr8, Chr12 and Chr15 each contained only one *CsLAC* member, and there were no *CsLAC* genes on Chr13. Moreover, some members of the *CsLAC* family on Chr4, Chr7, Chr9 and Chr10 exist in the form of gene clusters.

**Fig. 2:**
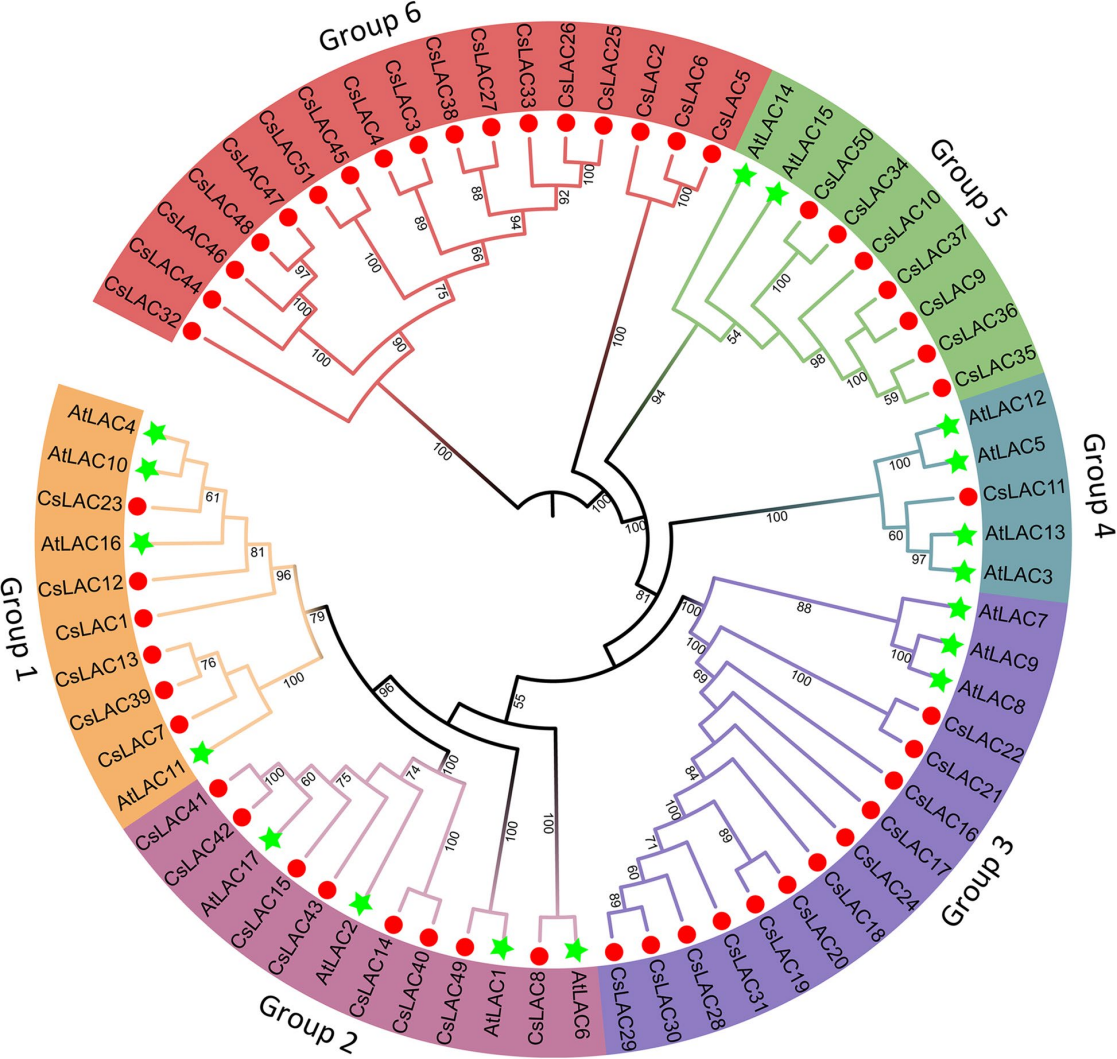
Phylogenetic analysis of *LAC* genes from *Arabidopsis* and *Camellia sinensis*. A phylogenetic tree was constructed with 17 *Arabidopsis* protein sequences and 51 *Camellia sinensis* protein sequences. A total of six subclades of the family are highlighted in distinct colours. Green pentacles and red circles represent the *LAC* genes from *Arabidopsis* and *Camellia sinensis*, respectively

To investigate the phylogenetic relationships of laccases between tea plant and *Arabidopsis*, we constructed a phylogenetic tree using the full-length protein sequences of 51 *CsLACs* and 17 *AtLACs*. Based on the classification standard of *Arabidopsis* laccases, 51 *CsLACs* were divided into six groups, and their distribution in each group was rather uneven (Fig. 2). In detail, six *CsLACs* were clustered with four *AtLACs* (*AtLAC4*, *AtLAC10*, *AtLAC11* and *AtLAC16*) in Group 1, Group 2 contained eight *CsLACs* and four *AtLACs* (*AtLAC1*, *AtLAC2*, *AtLAC6* and *AtLAC17*), twelve *CsLACs* were clustered with three *AtLACs* (*AtLAC7*, *AtLAC8* and *AtLAC9*) in Group 3, Group 4 contained only *CsLAC11* and four *AtLACs* (*AtLAC3*, *AtLAC5*, *AtLAC12* and *AtLAC13*), seven *CsLACs* were clustered with *AtLAC14* and *AtLAC15* in Group 5, and seventeen *CsLACs* were clustered without *AtLACs* in Group 6. The results revealed that *CsLAC* genes underwent specific evolutionary events after the divergence of tea plant and *Arabidopsis*.

### Homology analysis of the LAC gene family

**Fig. 3:**
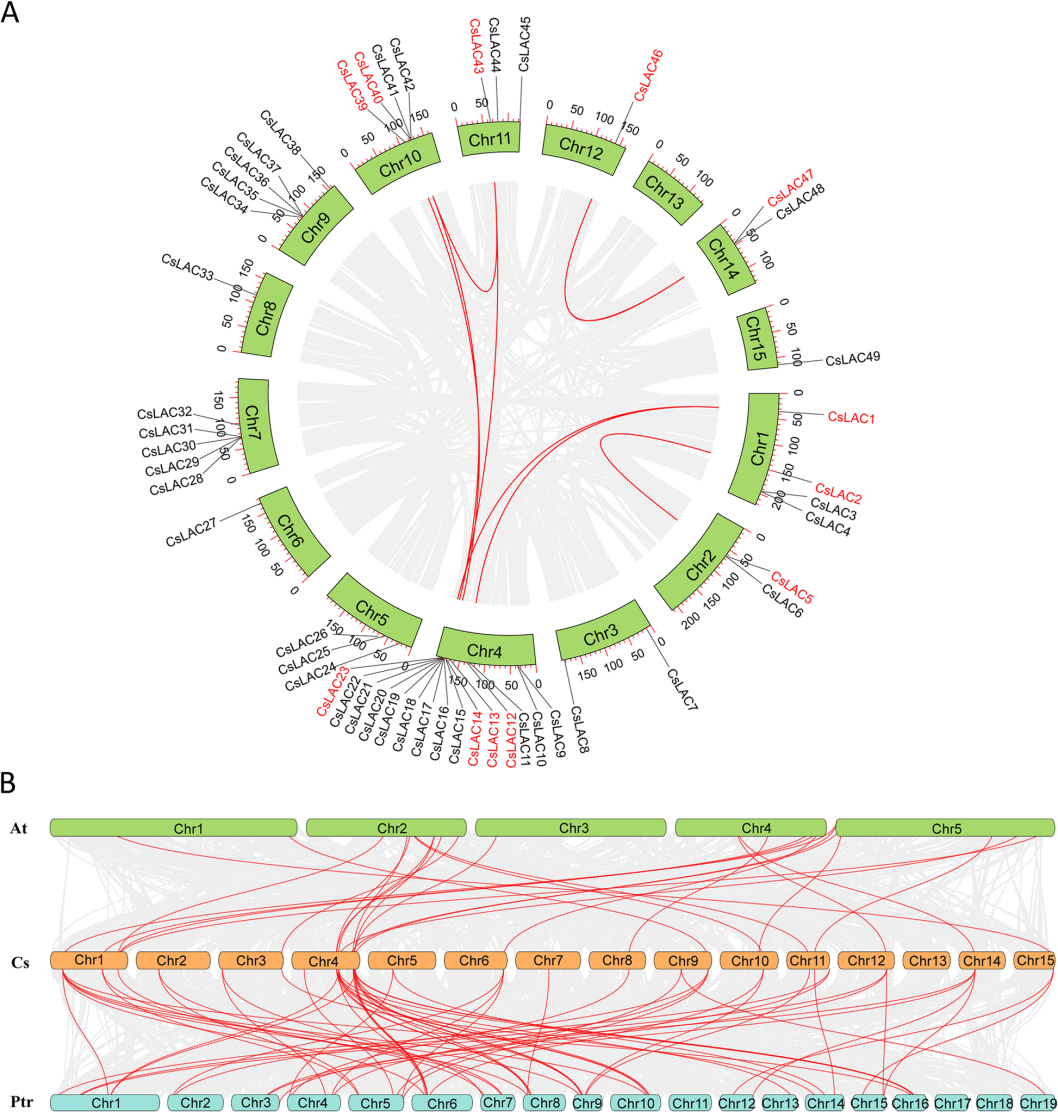
Collinearity of *LAC* gene pairs. (A) Collinearity analysis of the *CsLAC* gene family. All *CsLAC* genes were located on the chromosomes, and the identified *CsLAC* gene pairs are marked in red and connected by red lines. (B) Collinearity analysis of LAC genes across *Arabidopsis*, *Camellia sinensis* and *Populus trichocarpa*. The chromosomes of each species are represented by distinct colours, and the collinear gene pairs are connected by red lines

Gene duplication is considered to be one of the most important driving forces of genome evolution. Generally, gene duplication includes tandem repeats, segmental duplication and interspersed repeats, while segmental and tandem duplication are considered as the main factors of gene family expansion in plants. Studies have shown that the tea plant genome underwent two rounds of whole-genome duplication (WGD) events since they diverged from their common paleopolyploid ancestor [4, 5]. To investigate the gene duplication pattern of *CsLACs*, we performed collinear analysis. As a result, 30 out of 51 genes were tandem repeats, including 10 clusters of tandem repeat genes on eight chromosomes. Additionally, we found 16 *CsLAC* genes to be segmentally duplicated genes on seven chromosomes (Chr1, Chr2, Chr4, Chr10, Chr11, Chr12 and Chr14) (Fig. 3A).

To predict the function of *CsLACs*, we performed a homology analysis of *CsLAC* genes with *LAC* family genes from the model plant *Arabidopsis* and the woody model plant *Populus* (Fig. 3B). As a result, 25 homologous gene pairs were identified between *C. sinensis* and *Arabidopsis*, and 58 homologous gene pairs were obtained between *C. sinensis* and *Populus*.

### Motif compositions and gene structures

**Fig. 4:**
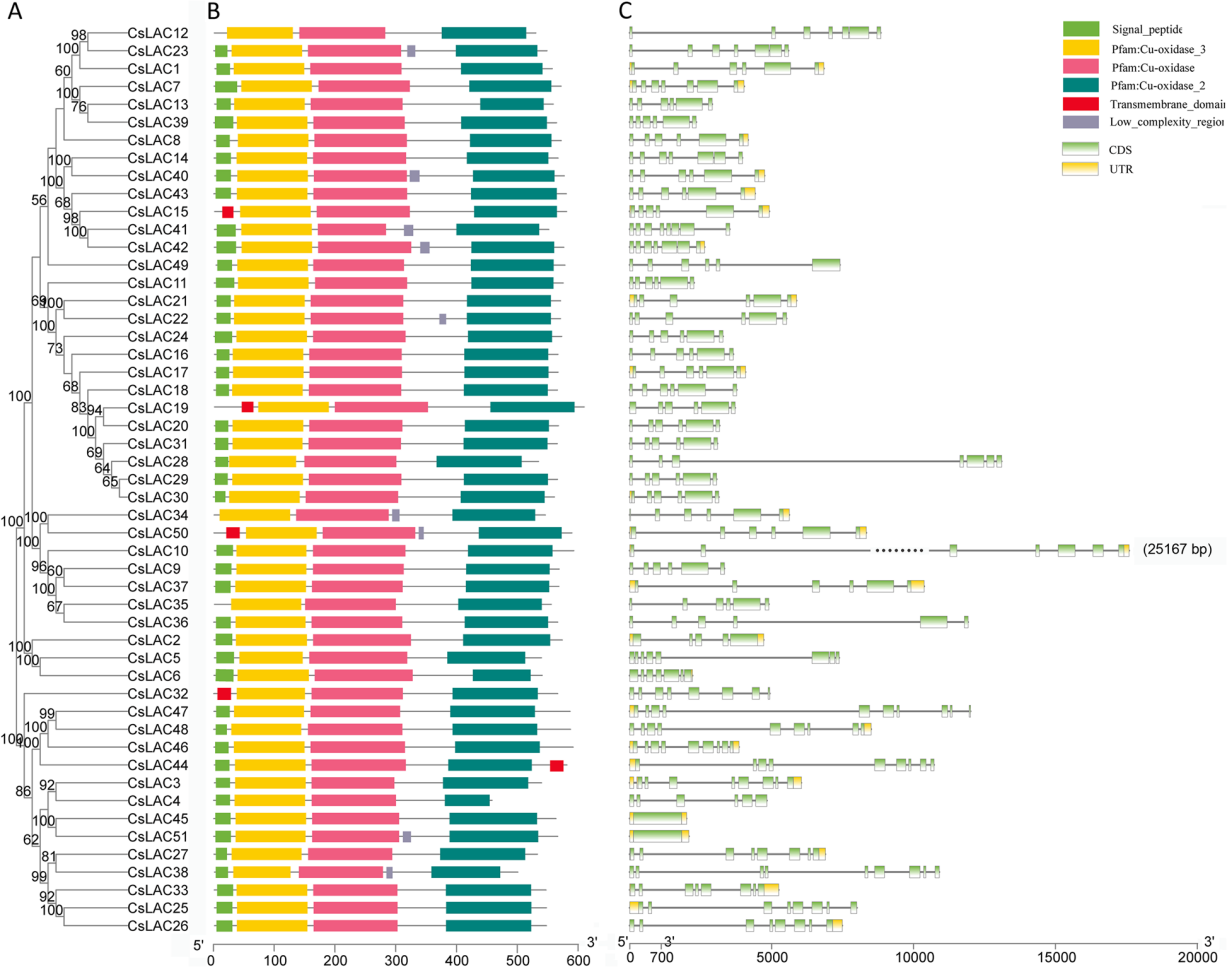
Phylogenetic tree, conserved domains and gene structure of *CsLACs*. (A) Phylogenetic relationship of *CsLACs*. (B) Conserved motifs and their distribution. The conserved motifs are named in the top-right corner and presented in different colours. (C) Gene structure of *CsLACs*. The UTR, CDS, and introns are represented by yellow boxes, green boxes and gray lines, respectively

We analyzed the 51 CsLAC proteins to reveal their conserved motifs using the MEME program and identified six types of motifs (Fig. 4B). As expected, all of the identified proteins contained three motifs (Cu-oxidase, Cu-oxidase_2, and Cu-oxidase_3). Many classes of CsLAC proteins had completely identical motif compositions, suggest that the possibility of functional redundancy among these genes. In addition, varying numbers or length differences of motifs across the CsLAC proteins may indicate functional divergence among some members.

To gain more insights into gene evolution, the exon‒intron organization of *CsLACs* was investigated by aligning coding sequences against their corresponding genomic sequences. The results showed that the gene structure of *CsLACs* exhibited diverse intron–exon patterns (Fig. 4C). For instance, except for both *CsLAC45* and *CsLAC51*, which had only one exon, the number of exons varied from 5 to 10 among the other *CsLAC* genes. Notably, *CsLAC10* had seven exons and was the longest gene with 25,167 bp in total. The *CsLAC* members with high homology had highly similar intron‒exon structures (intron number and exon length).

### Identification of cis-acting regulatory elements

**Fig. 5:**
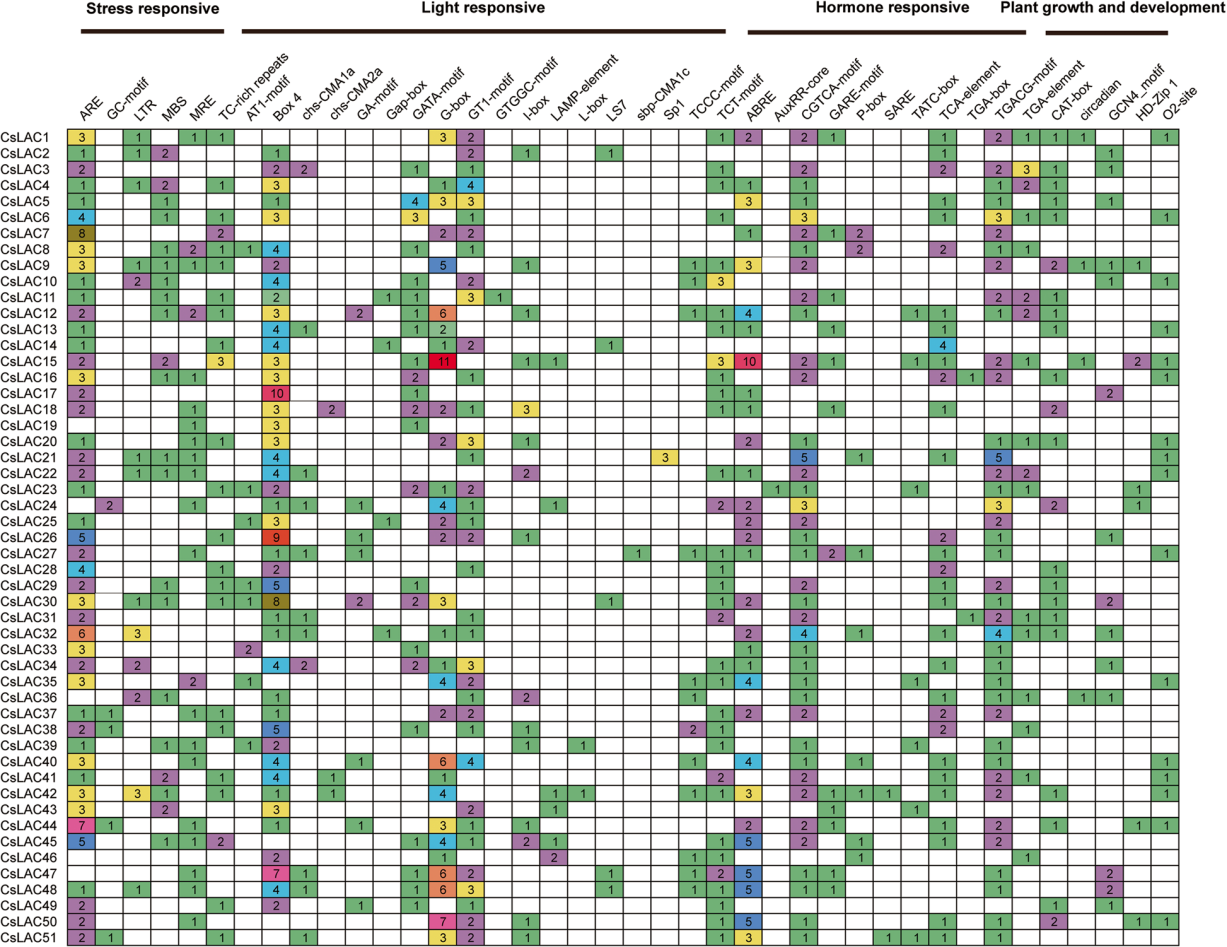
Identification of *cis*-acting elements of *CsLAC* genes. The distinct colours and numbers in the grid represent the numbers of different promoter elements in *CsLAC* genes

The *cis*-acting regulatory elements are located in the promoter region of target genes and can bind to appropriate transcription factors to regulate target gene expression in plants. To obtain insight into the regulation of *CsLAC* gene expression, we analyzed the *cis*-acting elements in the 2000 bp upstream sequences of the 51 *CsLAC* genes. A total of 40 types of *cis*-acting elements were obtained in the promoter regions of *CsLAC* family genes; these elements were divided into four categories, including stress responsive elements, light responsive elements, hormone responsive elements, and plant growth and development responsive elements (Fig. 5). Six types of elements belong to the stress‒responsive elements groups, including ARE (anaerobic inductive elements), GC-motif (enhancer-like element involved in anoxic specific inducibility), LTR (low temperature-inducible elements), MBS (MYB binding site involved in drought-inducible elements), MRE (MYB binding site involved in light responsiveness), and TC-rich repeats. The light‒responsive element group contained eighteen types of *cis*-acting elements; Box 4, GT1-motif, G-box and GATA-motif were relatively abundant among them. There were eleven types of *cis*-acting elements in the hormone responsive element group, and several important responsive elements were abundant, such as ABRE (abscisic acid-responsive element), CGTCA-motif (MeJA-responsive element), TCA-element (salicylic acid-responsive elements), TGACG-motif (MeJA-responsive element), and TGA-box (auxin-responsive element). The plant growth and development responsive element group included five types of *cis*-acting elements, such as CAT-box, circadian, GCN4-motif, HD-Zip1 and O_2_-site elements.

### Interaction network CsLAC proteins in tea plant

**Fig. 6:**
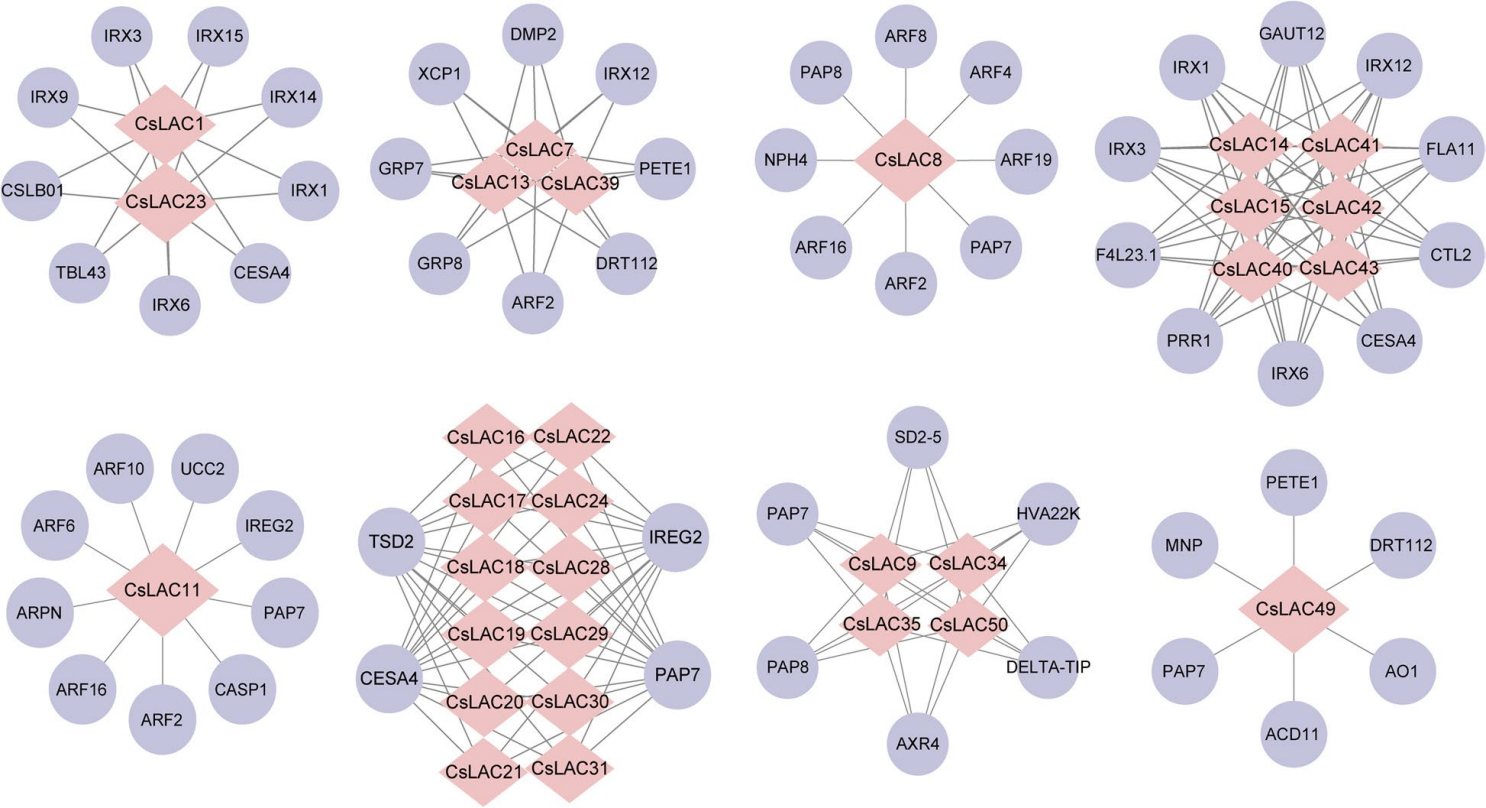
Interaction network of CsLAC proteins. There are 197 pairs of interacting proteins for 8 CsLAC subfamilies. The pink rhombus represents the CsLAC proteins in each subfamily; the purple circle indicates the interaction proteins in each clade

To investigate whether CsLAC proteins might function by forming homo- or hetero-protein complexes, we constructed a protein interaction network for CsLACs based on their orthology with AtLAC proteins (Fig. 6). A total of 197 interacting protein pairs were predicted in *Arabidopsis* and divided into eight subfamilies (Additional file 3: Table S3). The number and types of interacting proteins for each subfamily were obviously distinct. Nine proteins were predicted to interact with CsLAC1 and CsLAC23 proteins in the first subfamily; three members (CsLAC7, CsLAC13 and CsLAC39) had eight interacting proteins in the second subfamily; CsLAC8 had eight interacting proteins in the third subfamily; six members (CsLAC14, CsLAC15, CsLAC40, CsLAC41, CsLAC42 and CsLAC43) had ten interacting members in the fourth subfamily; CsLAC11 had nine interacting proteins in the fifth subfamily; twelve CsLAC proteins (CsLAC16, CsLAC17, CsLAC18, CsLAC19, CsLAC20, CsLAC21, CsLAC22, CsLAC24, CsLAC28, CsLAC29, CsLAC30 and CsLAC31) interacted with only four proteins in the sixth subfamily; four members (CsLAC9, CsLAC34, CsLAC35 and CsLAC50) in the seventh family had six interacting proteins; and only one member (CsLAC49) had six interacting proteins in the eighth subfamily.

### Expression patterns in different tissues

**Fig. 7:**
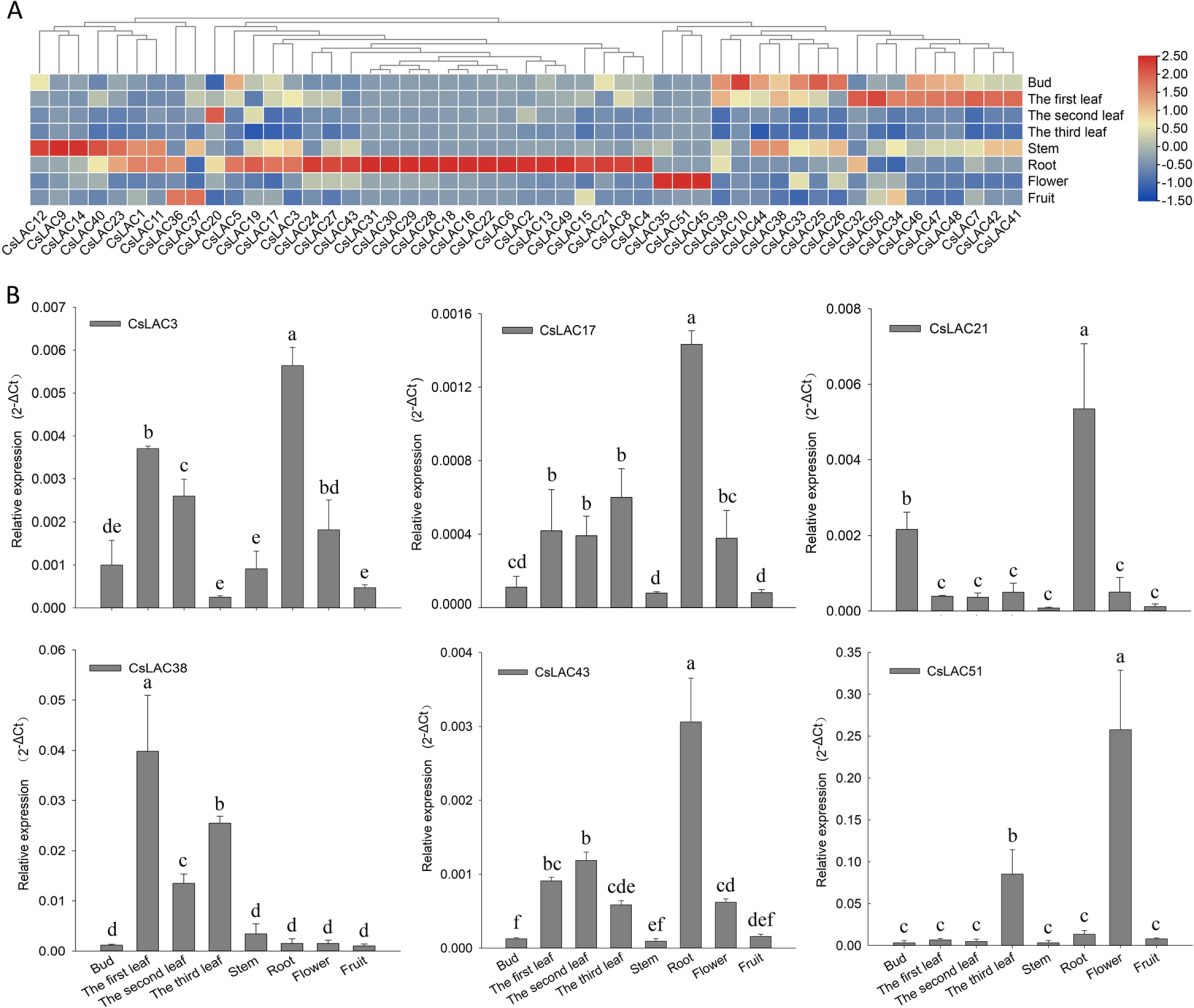
Expression profiles of *CsLAC* genes in eight different tissues. The eight tissues include the apical bud, the first leaf, the second leaf, the third leaf, budding flowers, young fruits, young roots and young stems. (A) Expression patterns of the 51 *CsLAC* genes in eight tissues based on mRNA-seq data. The colour scale on the right indicates log2 transformed TPM values, which represent high and low expression, respectively. (B) Expression levels of six genes in eight different tissues using qRT‒PCR. The expression values are the mean ± standard deviation of three independent biological replicates, and each biological replicate contained three technical replicates. Different letters above the bars denote significant differences at *P* < 0.05

To investigate the tissue-specific expression profiles of the *CsLAC* gene family, transcriptome data from eight distinct tissues were collected for further analyses. The tissue expression profiles were viewed in a heatmap, demonstrating that all *CsLAC* genes were detected in these tissues with diverse expression patterns (Fig. 7A). For instance, nine genes (*CsLAC1, CsLAC9*, *CsLAC11, CsLAC12*, *CsLAC14*, *CsLAC23, CsLAC38, CsLAC40* and *CsLAC44*) had extremely high expression levels in stems, twenty-four genes showed the highest expression level in roots (47.1%), three genes (*CsLAC35*, *CsLAC45* and *CsLAC51*) showed obviously higher expression levels in flowers than in the other tissues, and some genes had relatively higher expression levels in buds and leaves.

Based on the transcriptome data, we randomly selected six genes (*CsLAC3*, *CsLAC17*, *CsLAC21*, *CsLAC38*, *CsLAC43* and *CsLAC51*) for further validation of their expression patterns in eight different tissues by qRT‒PCR (Fig. 7B). As a result, the expression profiles of six genes based on qRT‒PCR were highly consistent with the results from transcriptome data. For instance, based on both qRT‒PCR and transcriptome data, four genes (*CsLAC3*, *CsLAC17*, *CsLAC21* and *CsLAC43*) had the highest expression level in roots, *CsLAC38* had a relatively higher expression level in leaves than in the other tissues, and *CsLAC51* showed a significantly higher expression level in flowers than in the other tissues.

### Expression patterns in response to drought and cold stresses

**Fig. 8:**
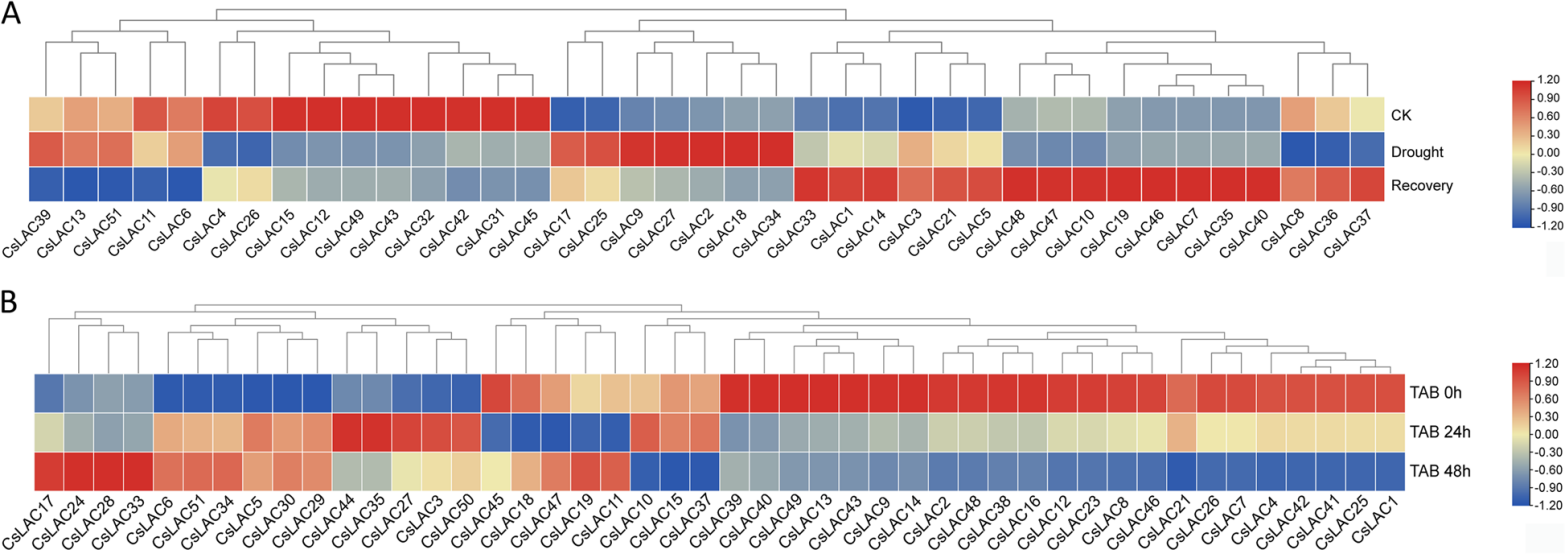
Expression profiles of *CsLAC* genes under drought and cold stresses. (A) A total of 39 *CsLAC* genes were distinctly expressed under drought stress compared to the control. (B) A total of 46 *CsLAC* genes were differently expressed under cold treatment compared to the control

Many studies have reported that *LAC* family genes participate in the response to abiotic stress, such as drought and cold stresses. Based on RNA-seq data, we analyzed the expression patterns of *CsLACs* under drought treatment and identified a total of 39 *CsLAC* genes (Fig. 8A). Five genes were dominantly downregulated under recovery after drought treatment, ten genes were significantly downregulated under drought and recovery treatments compared with the control, seven genes were significantly upregulated under drought and then downregulated after recovery, and seventeen genes had the highest expression level under recovery compared with the control and drought treatments.

We also analyzed the expression patterns of *CsLACs* under cold treatment based on transcriptome data. A total of 46 *CsLAC* genes were identified and showed diverse expression profiles (Fig. 8B). Four genes had significantly higher expression levels than the control after 48 h of treatment and at 24 h, six genes showed higher expression levels than the control at 24 and 48 h, five genes had the highest expression level at 24 h, and five genes showed the lowest expression level at 24 h.

### Expression patterns in response to insect and fungal stresses

**Fig. 9:**
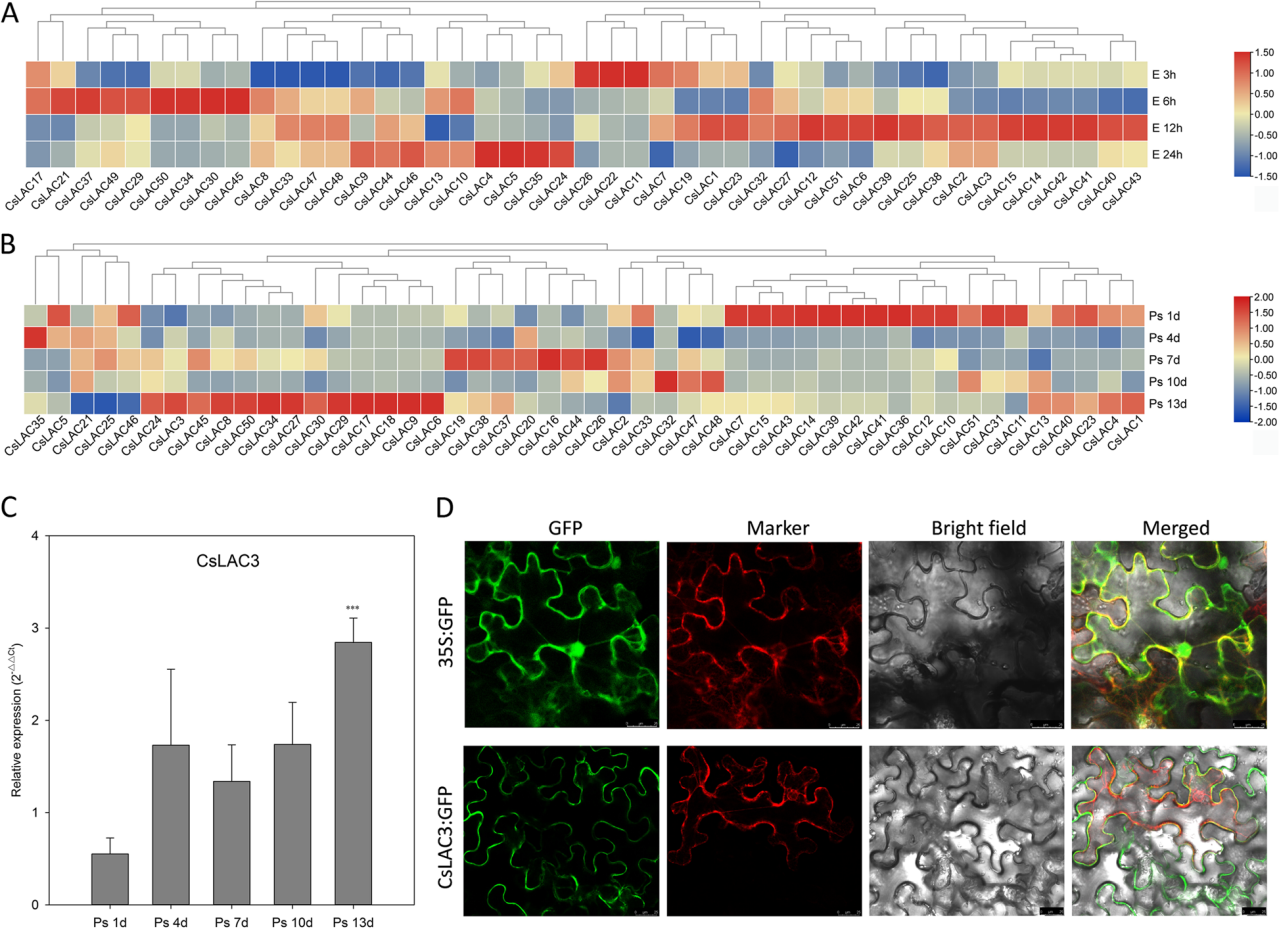
Expression profiles of *CsLAC* genes under *Ectropis obliqua* feeding and gray blight treatment and subcellular localization of *CsLAC3*. (A) A total of 45 *CsLAC* genes were identified with significantly different expression levels compared to the control. (B) A total of 48 *CsLAC* genes were significantly differentially expressed compared to the control. (C) Expression patterns of *CsLAC3* under gray blight treatment. The asterisks indicate the significant level (*** *P* < 0.001) based on a Student’s t-test. (D) Subcellular localization of the CsLAC3 protein. pCAMBIA1305 (empty vector) and pCAMBIA1305-CsLAC3 were transiently expressed in *Nicotiana benthamiana* leaves, scale bar = 25 μm

An analysis of *cis*-acting elements and the functional validation of *LAC* genes in different plant species demonstrated that *LAC* genes played an important role in response to biotic stresses, including fungal and insect pest disease. The expression patterns of *CsLACs* were analyzed under simulated *Ectropis obliqua* attack based on the RNA-seq data, and a total of 45 *CsLACs* were identified (Fig. 9A). Nine genes showed the highest expression level at 6 h after treatment, seven genes had higher expression levels at 24 h than at other time points, three genes (*CsLAC11*, *CsLAC22* and *CsLAC26*) displayed extremely high expression levels at 3 h, and eighteen genes had the highest expression level at 12 h time point.

A total of 48 genes were differentially expressed after gray blight treatment compared with the control (Fig. 9B). Only one gene (*CsLAC35*) had an extremely higher expression level at 4 d compared with the control and at other time points, eighteen genes showed significantly increased expression at 1 d after treatment, fifteen genes had the highest expression level at 13 d, seven genes showed the highest expression level at 7 d, and only three genes had the highest expression level at 10 d compared with the other four time points. Subsequently, we validated the expression pattern of *CsLAC3* under gray blight treatment (Fig. 9C). As a result, the expression level of *CsLAC3* decreased slightly at 1 d and increased significantly at 13 d, displaying a similar result as that found with the transcriptome data. To obtain insight into the molecular function of the CsLAC3 protein, we transferred the CsLAC3-GFP plasmid into *Agrobacterium* to infect tobacco leaves, and the results showed that the CsLAC3 protein was localized in the plasma membrane (Fig. 9D).

### Identification of cs-miR397a targeting CsLAC genes and their expression analysis in response to gray blight infection

**Fig. 10:**
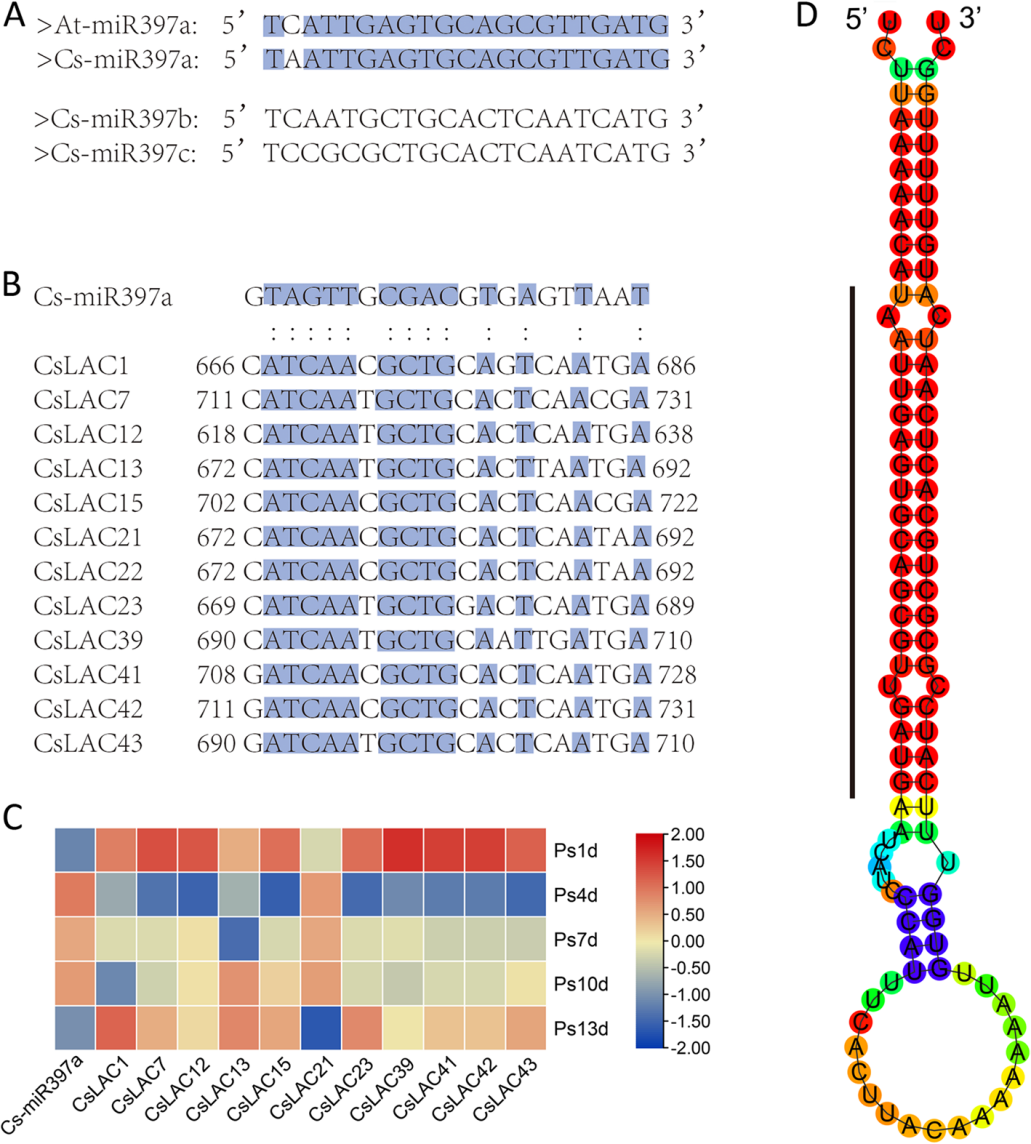
Putative miR397 target sites in *CsLAC* genes and the expression profile of ‘cs-miR397/*CsLACs*’ under gray blight disease stress

In plants, it was reported that *LAC* genes can be targeted and regulated by miR397 [39]. In tea plant, a total of four miR397 were identified based on previous studies [32, 40, 41], including cs-miR397a, cs-miR397b and cs-miR397c (Fig. 10A). To investigate the possible role of miR397 in regulating *CsLAC* genes, all 51 *CsLACs* were used to analyze the presence of potential target sites. As a result, 12 (*CsLAC1*, *CsLAC7*, *CsLAC12*, *CsLAC13*, *CsLAC15*, *CsLAC21*, *CsLAC22*, *CsLAC23*, *CsLAC39*, *CsLAC41*, *CsLAC42* and *CsLAC43*) out of 51 *CsLACs* were predicted to be the targets of cs-miR397a (Fig. 10B), while no *CsLAC* genes were targeted by cs-miR397b and cs-miR397c.

Moreover, the expression patterns of cs-miR397a and eleven predicted targets (except for *CsLAC22*) were analyzed under gray blight infection (Fig. 10C). The expression level of cs-miR397a was significantly downregulated at 1 and 13 d but upregulated at 4, 7 and 10 d. In comparison, most target genes (*CsLAC1*, *CsLAC7*, *CsLAC12*, *CsLAC15*, *CsLAC23*, *CsLAC39*, *CsLAC41*, *CsLAC42* and *CsLAC43*) showed opposite expression patterns. Furthermore, we cloned and sequenced pre-miR397a and obtained its double-stranded stem‒loop precursor structure (Fig. 10D). The results provide an important foundation for further investigating the role of ‘cs-miR397a/*CsLACs*’ in tea plants.

### Development and polymorphism analysis of SSR markers

A total of 36 SSR loci from 30 genes were obtained for designing primers. To test the reliability and polymorphism of these SSR loci, eight tea samples were selected for screening the primers. Among them, the markers without polymorphism of amplification, as well as those with ambiguous bands, were not used. As a result, a total of 18 SSR markers from 15 genes that generated both unambiguous and polymorphic bands were successfully developed. Subsequently, we selected 45 varieties/cultivars belonging to section *Thea* of the genus *Camellia* in the family Theaceae to test the tea plant germplasm resource transferability of these markers. The primer pairs of the 18 SSR markers and 45 tea samples are listed in Additional file 4: Table S4.

**Fig. 11:**
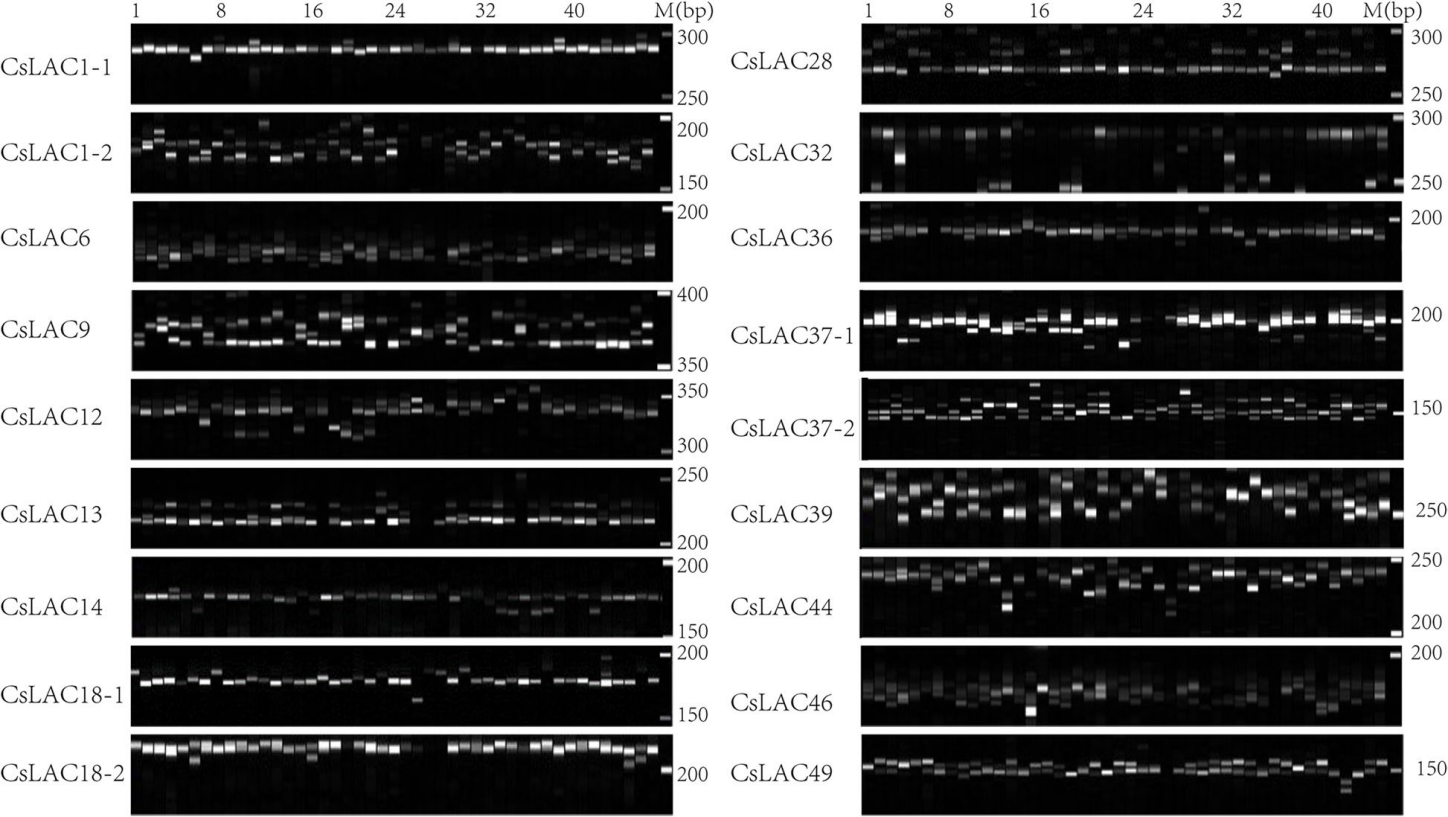
Gel electrophoresis image of 18 SSR markers among 45 tea varieties/cultivars

The majority of SSR markers displayed high polymorphism among the 45 tea samples, and the genetic properties of all the SSR markers were calculated (Fig. 11 and Table 2). The *Na* per locus ranged from 3 (CsLAC1-2, CsLAC6 and CsLAC39) to 8 (CsLAC49) with an average of 5.222 alleles. The *I* ranged from 0.468 (CsLAC1-2) to 1.598 (CsLAC36), with an average of 1.099. The *Ho* varied from 0.111 (CsLAC28) to 0.911 (CsLAC36), with an average of 0.510; the *He* ranged from 0.240 (CsLAC1-2) to 0.793 (CsLAC36), with an average of 0.566. The GD value ranged from 0.238 (CsLAC1-2) to 0.784 (CsLAC36), with an average of 0.560, and the PIC value varied from 0.221 (CsLAC1-2) to 0.750 (CsLAC36), with an average of 0.515. The results showed that these newly developed SSR markers from the *CsLAC* gene family are stable and highly polymorphic, providing a valuable resource for genetic research in tea plant.

**Table 2:** Characteristics of 18 developed SSR markers

Marker	*Na*	*I*	*Ho*	*He*	GD	PIC
CsLAC1-1	5	1.395	0.267	0.719	0.711	0.666
CsLAC1-2	3	0.468	0.178	0.240	0.238	0.221
CsLAC6	3	0.690	0.200	0.437	0.432	0.356
CsLAC9	6	1.506	0.711	0.725	0.717	0.685
CsLAC12	4	0.909	0.444	0.544	0.538	0.456
CsLAC13	7	1.223	0.733	0.578	0.572	0.539
CsLAC14	5	0.721	0.289	0.334	0.331	0.315
CsLAC18-1	5	1.306	0.689	0.680	0.672	0.630
CsLAC18-2	6	1.262	0.800	0.679	0.671	0.609
CsLAC28	4	0.559	0.111	0.263	0.260	0.248
CsLAC32	6	1.493	0.689	0.745	0.737	0.697
CsLAC36	6	1.598	0.911	0.793	0.784	0.750
CsLAC37-1	5	1.350	0.578	0.705	0.697	0.647
CsLAC37-2	6	1.232	0.600	0.649	0.642	0.582
CsLAC39	3	0.690	0.511	0.437	0.432	0.356
CsLAC44	5	0.967	0.667	0.561	0.554	0.469
CsLAC46	7	1.406	0.533	0.672	0.665	0.629
CsLAC49	8	0.999	0.267	0.423	0.418	0.406
Mean	5.222	1.099	0.510	0.566	0.560	0.515
St. Dev	1.437	0.355	0.239	0.172	0.717	0.685

Note: Na, the number of alleles; I, shannon’s information index; *Ho* observed heterozygosity, *He* expected heterozygosity; *GD* genetic diversity, *PIC* polymorphism information content; *St.Dev* standard deviation

## Discussion

Laccase enzymes are multicopper oxidases that play critical roles in the biosynthesis of lignin, which is involved in plant development and various stress responses. Systematic analyses have been conducted to identify laccase gene families in many model, crop and woody plants. Tea plant is one of the most important woody cash crops worldwide; however, there is little information about *CsLAC* genes. Here, a total of 51 *CsLACs* were identified based on the tea plant genome, and comprehensive analysis of the *CsLAC* gene family was performed. The number of *CsLAC* genes in tea plants is higher than that in most other plants studied, including *Arabidopsis thaliana* (17) [42, 43], *Brachypodium distachyon* (29) [16], *Oryza sativa* (30) [17], *Phyllostachys edulis* (23) [11], *Citrus sinensis* (24) [44], *Pyrus bretschneideri* (41) [19], and *Populus trichocarpa* (49) [45], while it is less than that in soybean (93) [23] and *Eucalyptus grandis* (54) [46]. For gene family number, tandem and segmental duplication events are the major reasons for gene expansion [47]. In tea plant, 49 *CsLACs* are unevenly distributed on 14 chromosomes and 2 *CsLACs* are on unassigned contigs, including 10 clusters of tandem repeat genes on eight chromosomes and 16 segmentally duplicated genes on seven chromosomes (Fig. 3A).

All 51 identified *CsLACs* had conserved copper-binding domains, while most of them had distinct gene structures, implying that they had similar genetic origins but had divergent biological functions. Notably, some transcription factors (TFs) may be involved in regulating the expression of *CsLAC* genes by the recognition of their *cis*-acting elements, such as G-box elements are generally exist in the promoters of light-responsive genes and can be bound by bZIP and bHLH TFs [48, 49], ABRE elements are often discovered in the promoters of ABA hormone‒responsive genes [50]. To understand the potential regulation of *CsLAC* expression, we analyzed *cis*-acting elements in the 51 *CsLAC* promoter regions. Four classes of *cis*-acting elements, including plant growth and development elements, stress responsive elements, light responsive elements and hormone responsive elements, were obtained. The putative *cis*-acting elements suggested that *CsLACs* function in various physiological processes, such as development, morphogenesis, and response to stresses. Additionally, tissue-specific expression profiles of the 51 *CsLACs* were analyzed, demonstrating that they had diverse expression patterns and preferred to a particular organ. The majority of *CsLACs* had the highest expression level in roots (47.1%), and nine *CsLACs* were preferentially expressed in stems (Fig. 7A). Similarly, most laccase genes are mostly expressed in roots and stems in several other plant species, such as *Arabidopsis* [42, 51], *Oryza sativa* [17], and *Eucalyptus grandis* [46]. Since both roots and stems contain a predominant amount of lignified tissues, these *CsLAC* genes might play important roles in lignin biosynthesis. It was shown that some *CsLACs* had predominant expression levels in buds and leaves, suggesting that they are involved in the growth and development of buds and leaves. Interestingly, *CsLAC35*, *CsLAC45* and *CsLAC51* had extremely high expression levels in flowers but lower to no-expression levels in other tissues; some genes were also found to be mainly expressed in flowers in other plant species including *Oryza sativa* [17], *Solanum melongena* [52], and *Phyllostachys edulis* [11]. The results indicate that these three *CsLAC* genes may play a major role in flower development.

Based on multiple sequence alignments, a phylogenetic tree containing 17 *AtLACs* and 51 *CsLACs* was constructed, and six groups were identified based on phylogenetic analysis (Fig. 2). *AtLAC4* and *AtLAC11* in Group 1 and *AtLAC2* and *AtLAC17* in Group 2 have been verified to be related to lignin biosynthesis [15, 20, 53], implying that *CsLACs* in the two groups are probably involved in lignin biosynthesis. In Group 4, twelve *CsLACs* were clustered with *Arabidopsis* laccases *AtLAC7*, *AtLAC8* and *AtLAC9*, which respond to environmental cues [42]. In Group 5, seven *CsLACs* were clustered with *AtLAC14* and *AtLAC15*, which have been reported to be involved in the polymerization of phenolic compounds [43, 54]. In upland cotton, *GhLAC1* and *GhLAC15* were phylogenetically related to *AtLAC14* and *AtLAC15*, which were participate in positively regulating defense-induced lignification to enhance the broad-spectrum biotic stress response [21, 55]. Based on the heatmap, all the *CsLACs* were involved in the response to herbivory feeding except *CsLAC36*, and the expression of seven *CsLACs* was positively regulated by fungal stress treatment (Fig. 9). Therefore, the *CsLACs* in Group 5 are probably involved in lignin biosynthesis and defense responses to biotic stresses. A total of seventeen *CsLACs*, but no *AtLACs,* were classified into Group 6, implying that these *CsLAC* genes may have distinct roles during tea plant evolution. *CsLAC3* in Group 6 was selected for further validation, displaying that CsLAC3 protein was localized in plasma membrane, had high expression in roots and leaves (Fig. 7) and was involved in the response to gray blight treatment (Fig. 9), while *CsLAC3* functions should be further validated.

Studies have shown that some LAC genes are targets of miR397, which is conserved across most plant species [56]. The plant miR397 family mainly targets *LAC* genes functioning in lignin biosynthesis and is involved in plant development and stress responses, such as floral organ and seed development, fruit development, drought and cold stresses, heavy metal stress, and pathogen stress [39]. We identified 12 *CsLAC* genes as potential targets of Cs-miR397a, which had only one base difference from At-miR397a (Fig. 10). Based on small RNA sequencing data, we analyzed the expression patterns of Cs-miR397 under cold [57], drought [58], insect herbivory [22], and fungal disease stresses [32]. However, no differentially expressed miR397 was identified under cold, drought and insect herbivory stresses, whereas Cs-miR397a was identified under gray blight infection. Under gray blight infection, the expression level of Cs-miR397a was downregulated at 1 and 13 d but upregulated significantly at 4, 7 and 10 d, while most of the potential *CsLAC* targets had the opposite expression patterns (Fig. 10C). In *Malus hupehensis*, it was reported that Mh-miR397b negatively regulates resistance to *Botryosphaeria dothidea* disease by modulating *MhLAC7,* which is involved in lignin biosynthesis. Therefore, we predicted that Cs-miR397a may be involved in fungal disease resistance by targeting *CsLACs* in tea plant, but further research is needed.

SSR molecular markers have gained considerable importance in plant genetic research due to their multiple-allelic nature, codominant inheritance, stability, and high abundance in the genome [37, 59]. After screening 36 SSR markers, we obtained 18 SSR markers that showed stable and unambiguous amplification bands in 45 tea samples (Fig. 11). The majority of SSR markers displayed high polymorphism with an average PIC value of 0.515, while CsLAC1-2 and CsLAC28 had low polymorphism with PIC values of 0.221 and 0.248, respectively. The polymorphism of SSR markers can be influenced by several factors, including the location of SSR loci in the genome, the number of markers, the sampling scheme, the accuracy of genotyping data, and the type of SSR motif repeats [37, 38]. In tea plant, several previous studies of genomic SSR marker development showed that the average PIC values for 13, 30 and 36 markers were 0.860, 0.704 and 0.862, respectively [38, 59, 60], while two studies showed that the average PIC values of SSR markers were similar to the average PIC value in our study [61, 62]. Overall, the newly developed SSR markers can be used for various genetic studies in tea plant, such as genetic variation, evolutionary origin, fingerprinting, QTL mapping, and marker-assisted selection breeding.

## Conclusions

In this study, we performed a genome-wide analysis of the *CsLAC* gene family, generated a wide range of expression data, including tissue-specific expression patterns and expression profiles of *CsLACs* responding to abiotic and biotic stresses, and developed some highly polymorphic SSR markers. This study provides target genes for regulating lignin biosynthesis in tea plant and lays the foundation for understanding the function of *CsLAC* genes.

## Supplementary Information

**Additional file 1:** **Table S1.** CsLAC gene family CDs and protein sequences. 

**Additional file 2:** **Table S2.** Primers developed for six CsLAC genes for qRT‒PCR and CsLAC3 for subcellular localization.

**Additional file 3:** **Table S3.** Proteins interacting with LAC proteins in Arabidopsis and C. sinensis. 

**Additional file 4:** **Table S4.** Primer pairs for 18 SSR markers and 45 tea plant samples used for SSR marker development.

## Data Availability

The data generated and analyzed in this study are included in this article and its Supplementary materials. RNA-Seq data of *Ectropis obliqua* feeding treatment are available at the NCBI SRA database (https://www.ncbi.nlm.nih.gov/) under project accession number PRJNA901518.

## Abbreviations

PAL: Phenylalanine ammonia-lyase
4CL: 4-(Hydroxy) cinnamoyl CoA ligase
C3H: P-coumarate 3-hydroxylase
C4H: Cinnamate 4-hydroxylase
CAD: Cinnamyl alcohol dehydrogenase
CCoAOMT: Caffeoyl CoA O-methyltransferase
HCT: Hydroxycinna-moyl-CoA:shikimate (SA)/quinate (QA) hydroxycinnamoyl transferase (HCT); CCR: Cinnamoyl CoA reductase
COMT: Caffeic acid/5-hydroxyferulic acid O-methyltransferase
F5H: Ferulate 5-hydroxylase
LAC: Laccase; TPM: Transcripts Per Million
SSR: Simple sequence repeat
